# Wolfberry (*Lycium barbarum*) Consumption with a Healthy Dietary Pattern Lowers Oxidative Stress in Middle-Aged and Older Adults: A Randomized Controlled Trial

**DOI:** 10.3390/antiox10040567

**Published:** 2021-04-07

**Authors:** Darel Wee Kiat Toh, Wan Yee Lee, Hanzhang Zhou, Clarinda Nataria Sutanto, Delia Pei Shan Lee, Denise Tan, Jung Eun Kim

**Affiliations:** Department of Food Science & Technology, Faculty of Science, National University of Singapore, 2 Science Drive 2, Singapore 117543, Singapore; dareltoh@u.nus.edu (D.W.K.T.); lee.wan.yee@u.nus.edu (W.Y.L.); hanzhang_zhou@u.nus.edu (H.Z.); e0254848@u.nus.edu (C.N.S.); lee.delia@u.nus.edu (D.P.S.L.); denise_tan@u.nus.edu (D.T.)

**Keywords:** antioxidant, body composition, *Lycium barbarum*, middle-aged, oxidative stress, plasma carotenoids, randomized controlled trial, skin carotenoids

## Abstract

Incorporating zeaxanthin-rich wolfberry (*Lycium barbarum*) into a healthy dietary pattern may augment its antioxidant potential. The present 16-week, parallel design randomized controlled trial aimed to investigate the impact of adhering to a healthy dietary pattern, either with or without whole dried wolfberry (15 g/d) on oxidative stress status (plasma malondialdehyde and 8-iso-prostaglandin F2α) in middle-aged and older adults. Changes to carotenoids status (plasma and skin carotenoids) and body composition were further evaluated to explore potential mechanisms which underlie the antioxidant properties of wolfberry. Plasma 8-iso-prostaglandin F2α, plasma zeaxanthin and skin carotenoids status were significantly raised in the wolfberry consuming group (*n* = 22; *p* < 0.05) compared to the control group which showed no changes (*n* = 18). Likewise in the wolfberry group only, inverse association was observed between the change values of plasma zeaxanthin and plasma 8-iso-prostaglandin F2α (−0.21 (−0.43, 0.00) ng/µmol, regression coefficient (95% CI); *p* = 0.05). Wolfberry consumption with a healthy dietary pattern may serve as a dietary strategy to attenuate lipid peroxidation among middle-aged and older adults who are at a heightened risk of oxidative stress induced age-related disorders. The antioxidant properties of wolfberry may be attributed to its rich zeaxanthin content.

## 1. Introduction

Declines in the endogenous antioxidant network are regarded as one of the few antagonistic hallmarks of aging [1]. This can be induced by mitochondrial perturbations which alter its functionality and makes the middle-aged and older population more vulnerable to oxidative stress [2]. Disruptions to the dynamic redox regulation results in oxidative damage and phenotypic changes which predisposes many age-related disorders such as cardiovascular disease, chronic obstructive pulmonary disease and dementia [3].

Under these circumstances, dietary antioxidants are especially important to support the maintenance of redox homeostasis. This may be attained through an adherence to healthy dietary patterns (HDPs), often characterized by a balanced consumption of fruits, vegetables, wholegrains and meats or alternative protein sources which contributes to a variety of exogenous antioxidants [4,5]. As supported by cross-sectional studies, inverse associations were reported between HDP compliance with plasma and urinary malondialdehyde (MDA) concentrations [6,7]. In addition, a meta-analysis evaluating randomized controlled trials (RCTs) deduced significant improvements in several oxidative stress parameters including blood MDA and glutathione after adherence to the Dietary Approaches to Stop Hypertension HDP, compared to the control [8]. On the contrary, while the intake of dietary antioxidants such as vitamin E and carotenoids display inverse associations with oxidative stress [9,10], inconsistent results were observed from clinical trials which intervened with antioxidant-rich foods or antioxidant supplementations [1,8,11,12]. This may be attributed to reasons such as functional concentration limits, poor bioavailability and the lack of antioxidant synergy in single antioxidant clinical trials [8,11,12,13]. Therefore, the incorporation of antioxidant-rich foods to a HDP may serve as a dietary strategy to augment its antioxidant potential [14].

Fruits of *Lycium barbarum* (wolfberry or goji berry), is one such example with exemplary antioxidant potential. Traditionally consumed in Asia for its medicinal and therapeutic properties [15], wolfberry has now become increasingly commercialized across the globe and incorporated as an ingredient into various culinary preparations [16]. Its benefits are thought to stem from bioactive compounds including carotenoids, polyphenols, vitamin C precursors and its *L. barbarum* polysaccharide (LBP) fraction [17].

In vitro and animal models which investigated the effects of wolfberry-based supplementations or extracts demonstrated its ability to not only scavenge reactive oxygen species (ROS) directly, but also, upregulate the expression and activity of endogenous antioxidant enzymes [18,19,20,21]. This largely aligns with previous RCTs. According to Amagase et al. [22], which intervened with wolfberry fruit juice (120 mL) for 30 days and Ma et al. [23] which administered LBP capsules (0.72 mg LBP) for 6 weeks, reductions in MDA concentration, coupled with a raised production of antioxidant enzymes such as superoxide dismutase and glutathione peroxidase in blood were reported in healthy subjects.

Despite the emerging evidence which suggests the antioxidant benefits of wolfberry, limited studies explored its effects as whole dried fruits or considered possible synergism with a HDP. This is especially so amongst middle-aged and older adults who are more susceptible to oxidative stress. Therefore, the objective of the present RCT is to investigate the impact of dietary counselling (to adhere to a HDP), either with or without the incorporation of whole dried wolfberry on oxidant burden (plasma MDA and 8-iso-prostaglandin F2α (8-isoPGF2α)) in a middle-aged and older Singaporean population. Changes to the participants’ carotenoids status (plasma carotenoids and skin carotenoids status (SCS)) and body composition were further explored to define the mechanisms which underlie the potential antioxidant effects of wolfberry.

## 2. Materials and Methods

### 2.1. Study Design and Participants

The present study was a parallel design, 16-week RCT with 2 intervention arms and 5 study visits in total to the National University of Singapore and National University Hospital. It was conducted between July 2018 and October 2019, in Singapore. The protocol was approved by the National Healthcare Group Domain Specific Review Board and was registered at clinicaltrials.gov as NCT03535844. Written informed consent was obtained from prospective participants (*n* = 88), who were screened based on a set of criteria established *a priori*.

The screening encompassed an interview and structured questionnaires designed to obtain details on the participants’ sociodemographic and lifestyle characteristics, medical history as well as their habitual dietary patterns. To be eligible, participants should be aged ≥50 and ≤75 years, and have sufficient venous access for blood draw. Exclusion criteria included those who were smokers; prescribed long-term medications (e.g., anti-hypertensives, glucose or lipid lowering drugs) five years prior; taking dietary supplements or traditional Chinese medicine one month prior; experiencing ±5% body weight changes three months prior; consuming >3 and >2 alcoholic beverages/week for males and females respectively; exercising vigorously (defined as having >6 metabolic equivalents daily [24]); pregnant or lactating; exposed to radiation <3 months prior; or were habitually compliant to the “My Healthy Plate (MHP)”, a HDP recommended by the Singapore Healthy Promotion Board.

**Figure 1:**
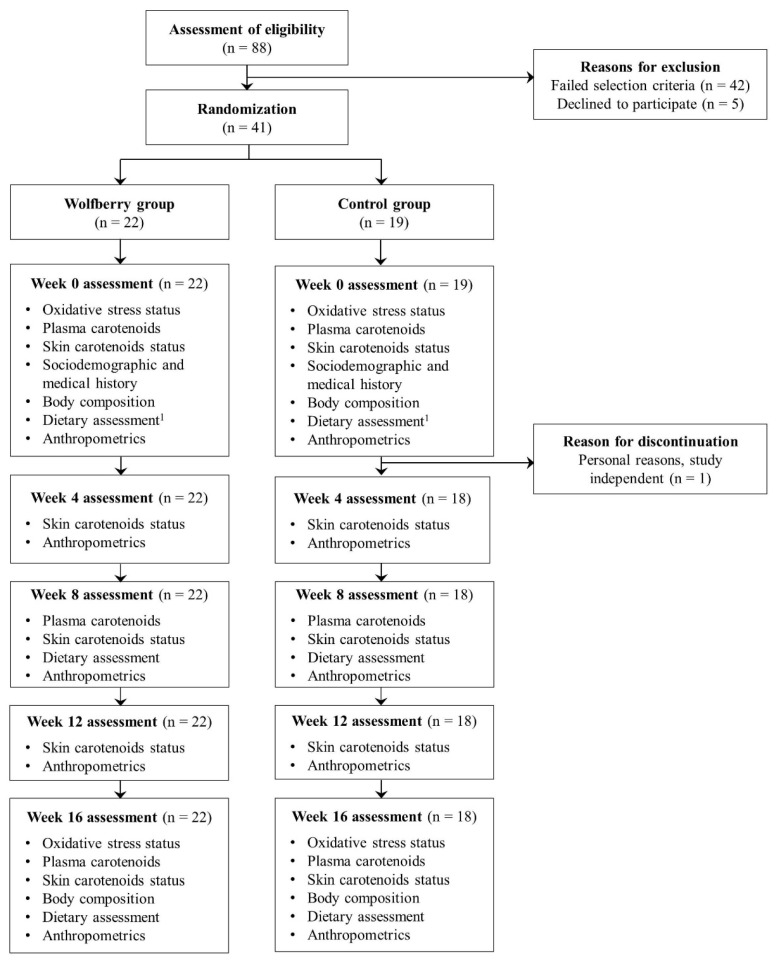
Consolidated Standards of Reporting Trials (CONSORT) flow diagram for randomized controlled trial ^2^. ^1^ Dietary assessment was absent for one participant from each group at week 0. ^2^ The wolfberry group received dietary counselling to adhere to a healthy dietary pattern and consumed 15 g/d whole while the control group received dietary counselling to adhere to a healthy dietary pattern only.

Enrolled participants (*n* = 41) were randomly assigned to the wolfberry (*n* = 22) and control (*n* = 19) groups using STATA/MP 13′s (StataCorp LP, College Station, TX, USA) random number function. The randomization work was conducted by a research staff, independent of the enrollment, investigation and formal analysis. Each participant’s allocation was concealed until the week 0 assessments were completed. From the participants, 1 withdrew due to personal reasons independent of the study while the remaining 40 participants attended all 5 study visits and completed the intervention. The habitual dietary data at week 0 was absent for 1 participant from each group. A Consolidated Standards of Reporting Trials (CONSORT) flow diagram which further elaborates on the participant flow and outcome variables examined during each study visit is summarized in Figure 1.

### 2.2. Interventions and Compliance

Dietary counselling to follow the MHP was administered by trained research nutritionists to all participants at week 0. For adults ≥ 51 y, the MHP daily recommendations comprise of four to six servings of wholegrains (e.g., brown rice, whole wheat bread, oatmeal etc.), two servings of fruits and two servings of non-starchy vegetables (starchy vegetables include potatoes, tapioca, yam etc.), and three servings of non-processed meats and alternative proteins. Details of the dietary pattern are more extensively elaborated in a fact sheet by the Singapore Health Promotion Board [25]. Specific to the wolfberry group, instructions were given to cook (by steaming or steeping in hot water) and consume 15 g of whole dried wolfberry (Ning Xia, China; purchased from Eu Yan Sang Pte. Ltd., Singapore) with their main meals. This included the consumption of both the whole fruit and residual liquid concoction daily, throughout the 16-week study duration. Double-blind was challenging in the present context due to the nature of the intervention. However, main study investigators involved in outcome assessment, data curation as well as formal and statistical analyses were blinded to the treatments.

Compliance to the dietary intervention was determined for each participant using 3-day food records and single day photographic records every 4 weeks. Recording procedures (i.e., attention to product detail, quantity consumed and mode of preparation etc.) were carefully instructed by research nutritionists who reviewed the submitted 3-day food records in detail each visit to improve dietary data accuracy and precision. Shortcomings to the MHP during each visit were also identified for tailored dietary counselling to optimize MHP conformity. The food records were analyzed using DietPlan 7 (Forestfield Software Ltd., Horsham, West Sussex, UK) with the US Department of Agriculture (USDA) National Nutrient Database for Standard Reference, Release 27, as the primary database [26]. This was complemented and cross-referenced with secondary data sources which comprised of the Singapore Health Promotion Board nutrient composition database for local cuisines [27] and nutritional information panels for commercial food products.

### 2.3. Outcome Variable Measurements

After an overnight fast (>10 h), venous blood was collected into EDTA-coated vacutainer tubes (Becton Dickinson, Franklin Lakes, NJ, USA) for centrifugation (3000× *g* for 15 min at 4 °C). The plasma supernatant was stored as aliquots (500 μL) at −80 °C until analysis.

Plasma samples were used to evaluate the oxidant burden which served as the present study’s primary outcome. Secondary outcomes included the participant’s plasma carotenoids concentration and SCS, as well as their corresponding dietary carotenoids intake, body composition and anthropometric measurements.

#### 2.3.1. Biomarkers of Oxidative Stress

Indexes of lipid peroxidation, plasma MDA and 8-isoPGF2α, were selected as biomarkers of oxidative stress. MDA was analyzed using a Parameter assay kit (R&D Systems, Minneapolis, MN, USA) by reaction between acid-treated plasma with thiobarbituric acid. After incubation (50 °C, 3 h), optical densities were assayed spectrophotometrically with a plate reader at 532 nm (BioTek Instruments, Inc., Winnoski, VT, USA).

Plasma samples were prepared in accordance to the manufacturer’s instructions for 8-isoPGF2α analysis using enzyme-linked immunosorbent assay (ELISA; ABCAM, Cambridge, UK). In brief, acidified plasma samples (glacial acetic acid to pH 4.0) were first vortexed with ethyl acetate and centrifuged (250× *g* for 10 min at 22 °C). The organic layer was pooled thrice to isolate 8-isoPGF2α before it was vacuum dried (SpeedVac^®^, Thermo Fisher Scientific, Waltham, MA, USA) and re-dissolved in ethanol and a sample dilution buffer for ELISA. Solvents used were analytical grade and obtained from VWR International (Radnor, PA, USA).

#### 2.3.2. Plasma and Skin Carotenoids Status

**Figure A1:**
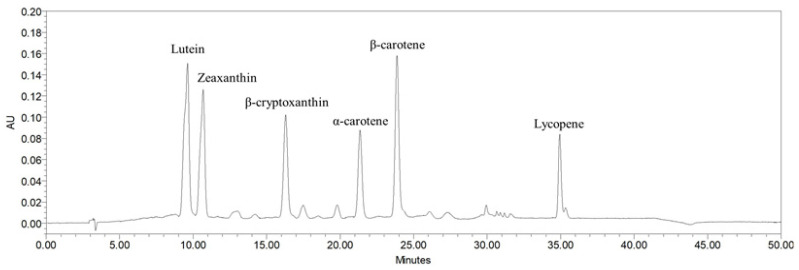
Representative chromatogram for plasma carotenoids analysis.

Plasma carotenoids were extracted and analyzed as previously described [28]. In brief, plasma aliquots were deproteinated and extracted with methanol, acetone and petroleum ether before reconstitution in methanol:methyl tert-butyl ether (MTBE) (1:1, vol:vol). Samples were analyzed using high performance liquid chromatography (HPLC) equipped with a C_30_ column (YMC, Kyoto, Japan) and photodiode array detector (Waters Alliance e2695 Separation Module; Waters, Milford, MA, USA) to resolve and quantify β-carotene, α-carotene, β-cryptoxanthin, lutein, zeaxanthin and lycopene. With the exception of lycopene (470 nm), the remaining carotenoids were detected at 450 nm. Chemicals for extraction and HPLC analysis were HPLC grade and pure carotenoids standards were analytical grade (≥95% purity). Acetone, methanol and petroleum ether were obtained from VWR International (Radnor, PA, USA) while MTBE and individual carotenoid standards were obtained from Sigma-Aldrich (St. Louis, MO, USA). A representative chromatogram is depicted in Figure A1 in the Appendix A.

SCS was measured using a Pharmanex BioPhotonic Scanner S3 (Nu Skin Enterprises, Provo, UT, USA) by resonance Raman spectroscopy, in duplicate. Each palm scan yielded a score from 10,000 to 89,000, which was directly correlated to the carotenoids content in the skin.

#### 2.3.3. Anthropometrics and Body Composition

Height and weight were measured using a stadiometer and digital scale (Seca, Hamburg, Germany) with the body mass index (BMI) calculated. Measurements of waist circumference were performed using a flexible tape measure at standing position after several consecutive natural breaths. This was in accordance to WHO standards, along the mid axillary line, between the top of the iliac crest and last rib [6]. Anthropometric measurements were conducted in duplicate.

Body composition analysis with whole body dual energy x-ray absorptiometry (DXA) scans was acquired with Hologic Discovery Wi, fan beam x-ray bone densitometer (Hologic Inc., Marlborough, MA, USA) in accordance to the protocol described by the National Health and Nutrition Examination Survey (NHANES) [29]. The scan results were analyzed to yield measures of the fat mass and lean mass and their distributions in the truncal and appendicular regions.

### 2.4. Power Calculation and Statistical Analysis

Power calculations (G*Power 3.1, Heinrich-Heine-Universität, Düsseldorf, Germany [30]) were conducted *a priori* based on a previous RCT which administered 14 g wolfberry/d and reported significant reductions in plasma thiobarbituric acid reactive substances (TBARS; wolfberry intervention group: −3 ± 1 μmol/mg of protein; control group +3 ± 1 μmol/mg of protein) after 45 days [12]. At a power of 90% and significance level of 0.05 (two-tailed), 16 subjects per group would be required to confirm a statistical difference, presuming the present study yields a similar response as previously.

Statistically, between-group differences at week 0 were first evaluated with independent t-tests and Fisher’s exact test for continuous and categorical variables respectively. Changes in all outcome variables (i.e., biomarkers of oxidative stress, carotenoids status, anthropometrics and body composition) across various time points were analyzed with two-way mixed model ANOVA. A post-hoc simple effects test by multiple contrasts of Bonferroni was used to further examine changes from week 0 within each group. For outcome variables which were significantly different between groups at week 0, the aforementioned statistical analysis was repeated with normalized values, expressed as a percentage change from week 0. This was coupled with an analysis of covariance (ANCOVA) between week 16 outcomes values, adjusted for the corresponding week 0 values for a more robust statistical evaluation [31]. A similar ANCOVA model was likewise used to compare dietary data between groups at weeks 8 and 16.

To examine the relationships between the change values of plasma carotenoids, SCS and body composition indicators with the corresponding changes in the biomarkers of oxidative stress, simple linear regression (SLR) and multiple linear regression (MLR; controlled for age, sex and BMI) were used. Variables which influenced plasma carotenoids and SCS, independent of dietary carotenoids, were selected as covariates for the MLR model. This was defined based on an earlier cross-sectional study by the same research group [28]. The regression analyses were performed by intervention group, as well as for combined datasets.

Statistical tests were conducted on STATA/MP 13 (StataCorp LP, College station, TX, USA) with a statistical significance of 0.05, two-tailed. All data are presented either as mean (SD) or β-coefficient (95% confidence intervals (CI)) unless otherwise stated.

## 3. Results

### 3.1. Participant Characteristics

The present RCT recruited both middle-aged and older (range: 50 to 64 years) males (wolfberry: *n* = 4, control: *n* = 7) and females (wolfberry: *n* = 18, control: *n* = 11). At week 0, demographic characteristics (age and sex) as well as the biomarkers of oxidative stress, anthropometrics and body composition indicators were similar between the wolfberry and control groups (*p* > 0.05). This is with the exception of plasma total carotenoids, specifically plasma lycopene, which was significantly higher in the control group (total carotenoids: 2.75 (0.95) µmol/L; lycopene: 0.58 (0.45) µmol/L) than the wolfberry group (total carotenoids: 2.02 (0.90) µmol/L; lycopene: 0.33 (0.22) µmol/L) (Table A1 in the Appendix A).

### 3.2. Dietary Assessment and Compliance

At week 0, an assessment of individual food groups indicated no differences apart from wholegrain intake which was significantly higher in the control group than the wolfberry group (wolfberry: 0.3 (0.4) servings/d; control: 1.0 (0.8) servings/d, *p* = 0.001) (Table A2 in the Appendix A). Nonetheless, at weeks 8 and 16, a similar extent of compliance was attained for wholegrains, vegetables, meats and alternatives (*p* > 0.05). While a marked discrepancy in fruits intake was observed at week 16 (wolfberry: 3.4 (1.4) servings/d; control: 2.4 (1.2) servings/d, *p* = 0.028), this was null following the exclusion of wolfberry which contributed to an additional 0.5 servings/d (*p* = 0.24 with wolfberry exclusion).

Good compliance to the MHP was observed for fruits, vegetables, meats and alternatives intake which largely met the 2, 2 and 3 servings/d recommendations respectively across both groups (Table A2). This is with the exception of wholegrains which increased but fell short of the 4 servings/d recommendations (wolfberry: 2.2 (2.3) servings/d; control: 1.8 (1.0) servings/d). For the dietary carotenoids, lycopene intake was markedly higher in the wolfberry group (wolfberry: 0.27 (0.21) mg/d; control: 0.41 (0.27) mg/d, *p* = 0.011) at week 16 only. The remaining dietary carotenoids, however, depicted no differences between groups at weeks 0, 8 and 16 (*p* > 0.05). There were no adverse effects observed in either group after intervention.

### 3.3. Oxidative Stress and Carotenoids Status

**Figure 2:**
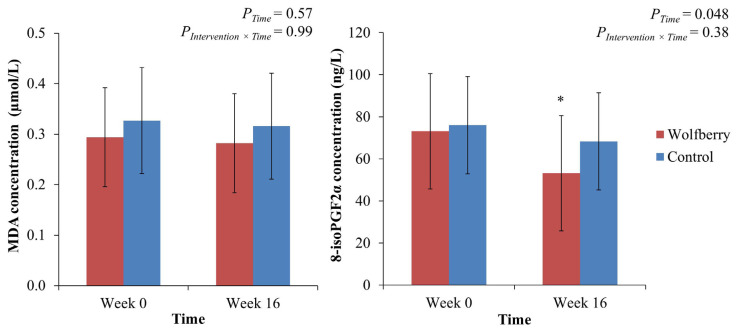
Concentrations of plasma oxidative stress biomarkers MDA and 8-isoPGF2α for wolfberry group (*n* = 22) and control group (*n* = 18) of randomized controlled trial. Values are means with error bars representing the SD. Data analyzed with two-way mixed model ANOVA and post-hoc simple effects test by Bonferroni’s multiple contrasts. * *p* < 0.05 compared to the corresponding week 0 values. At week 0, there were no significant differences in plasma MDA (*p* = 0.42) and 8-isoPGF2α (*p* = 0.82). Abbreviations: MDA, malondialdehyde; 8-isoPGF2α, 8-iso-prostaglandin F2α.

Changes to the biomarkers of oxidant burden, plasma MDA and 8-isoPGF2α are presented in Figure 2. While, MDA depicted no changes (wolfberry: 0.29 (0.10) to 0.28 (0.10) µmol/L; control: 0.33 (0.16) to 0.32 (0.11) µmol/L), a significant time effect (*p* = 0.048) was observed for 8-isoPGF2α (wolfberry: 73.1 (49.1) to 53.2 (27.4) ng/L; control: 76.0 (24.8) to 68.3 (23.1) ng/L). This was primarily driven by the wolfberry group which depicted a marked decline after intervention, at week 16.

Significant time (*p* = 0.041) and interaction (intervention × time) effects (*p* = 0.008) were observed for plasma total carotenoids (Table 1), largely attributed to the prominent rise in the wolfberry group at week 8 (+51.1 (112.1) % from week 0). This corresponds to the significant interaction effects observed for plasma β-carotene (*p* = 0.029), α-carotene (*p* = 0.034) and zeaxanthin (*p* < 0.001) which were likewise raised in the wolfberry group at week 8.

Specifically, only the increase in plasma zeaxanthin remained significant until week 16 in the wolfberry group (+62.5 (92.4) % from week 0), also an indicator of good compliance to wolfberry consumption. The remaining carotenoids depicted no changes although it was noted that in the control group, plasma β-cryptoxanthin (−31.6 (36.8) % from week 0) and lycopene (−43.6 (36.9) % from week 0) were markedly lowered at week 16.

The significant differences observed with normalized percentage change values were likewise reflected when similar statistical tests were conducted with absolute plasma carotenoids concentrations (Table A1). Upon adjustment of week 0 concentrations, comparisons between week 16 plasma carotenoids concentrations further suggests that among the six carotenoids, only zeaxanthin was identified to be higher in the wolfberry group, than the control group after intervention.

**Figure 3:**
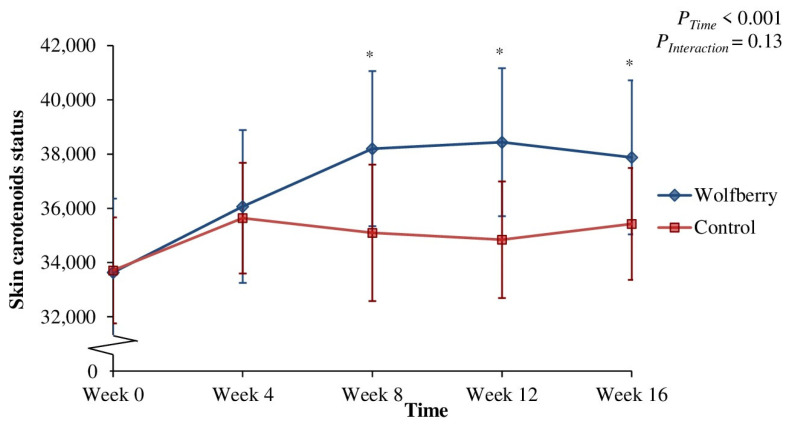
Skin carotenoids status for wolfberry group (*n* = 22) and control group (*n* = 18) at 4-week intervals of randomized controlled trial. Values are means with error bars representing the corresponding standard errors of mean. Data analyzed with two-way mixed model ANOVA and post-hoc simple effects test by Bonferroni’s multiple contrasts. * *p* < 0.05 compared to the corresponding week 0 values. Skin carotenoids status was not significantly different between groups at week 0 according to independent t-tests (*p* = 0.98).

Figure 3, which depicts the time course changes in SCS, indicated a significant time effect (*p* < 0.001). This is contributed by the wolfberry group which reported a significantly higher SCS from week 8 until week 16.

### 3.4. Associations between Changes in Oxidative Stress and Carotenoids Status

Regression analyses which examined the correlations between the biomarkers of oxidative stress with plasma carotenoids and SCS are described in Table 2. In the wolfberry group, significant inverse associations were observed between the change values of plasma 8-isoPGF2α with the corresponding changes in plasma β-cryptoxanthin (SLR coefficient (SLR β): −0.319 (−0.549, −0.089) ng/µmol; MLR coefficient (MLR β): −0.305 (−0.555, −0.054) ng/µmol) and zeaxanthin (MLR β: −0.213 (−0.428, 0.002) ng/µmol). In the control group, inverse associations exists for β-carotene (SLR β: −0.610 (−1.089, −0.132) ng/µmol; MLR β: −0.807 (−0.136, −0.259) ng/µmol) and SCS (MLR β: −0.004 (−0.007, −0.001) ng/µmol). Further evaluation using combined data indicated significant negative associations for a majority of plasma carotenoids including the concentrations of total carotenoids, β-carotene, α-carotene, β-cryptoxanthin, lutein and zeaxanthin (*p* < 0.05; Table A3 in the Appendix A).

Consistently, changes in plasma MDA showed no correlation with change values for both plasma carotenoids concentrations and SCS across all regression models (Table A3).

### 3.5. Anthropometrics and Body Composition

The effects of intervention on anthropometry and body composition are shown in Table 3. No clear changes were observed for BMI and waist circumference across both groups. Broadly however, for both appendicular and truncal regions, indicators of fat mass and fat percentage were reduced while indicators of lean mass were raised in both groups although the change was non-significant. Notable time induced effects were observed for total lean mass (*p* = 0.016) and truncal lean mass (*p* = 0.014) which were both higher, especially within the control group after 16 weeks.

Between the change values for body composition and the corresponding changes to lipid peroxidation biomarkers, associations were present in neither the wolfberry nor control groups. (Table A4 in the Appendix A). 

## 4. Discussion

Oxidative stress susceptibility increases with age due to a reduced efficacy of the endogenous antioxidant system [2]. While this may be strengthened by exogenous factors such as the adherence toward a HDP, the consumption of wolfberry, which is rich in dietary antioxidants, may further strengthen defense against oxidative stress [32]. The findings from our current RCT indicated that the incorporation of whole dried wolfberry to the MHP lowers oxidant burden in middle-aged and older adults over a 16-week intervention period. The inverse association between the change values for plasma 8-isoPGF2α and plasma zeaxanthin further suggests that the rich zeaxanthin content in wolfberry may be partly responsible for the alleviation of oxidative stress. Although there were improvements in lean mass following adherence to the MHP, wolfberry intake did not confer any further benefits to body composition.

Oxidative stress in the present study was determined by examining the products of free radical catalyzed polyunsaturated fatty acid peroxidation, MDA and 8-isoPGF2α. Specific to plasma 8-isoPGF2α, a marked reduction in oxidant production was detected following wolfberry intake only. This aligns with the results from earlier an RCT [33], which reported an increase in plasma antioxidant capacity after a 3-month intervention with 13.7 g/d of a milk-based wolfberry formulation in older adults [33]. Similarly, in another 8 week-RCT, consuming an aqueous concoction prepared with 13.5 g wolfberry daily significantly lowered oxidative stress induced lymphocyte DNA damage [34]. Epidemiological evidence, including a systematic review, consistently demonstrated direct associations between 8-isoPGF2α concentrations and chronic diseases incidence. Retrospective inspection of case-control models indicated that cases, which included individuals with hypertension, coronary artery disease and type 2 diabetes diagnosis, have approximately 10%, 28% and 30% higher plasma 8-isoPGF2α than controls respectively [35,36]. Furthermore, in the Coronary Artery Risk Development in Young Adults (CARDIA) study, it was deduced that a 1 SD increment in 8-isoPGF2α increases the odds of coronary artery calcification by 24% [37]. Although caution in interpretation is required, the present 37% decrease in 8-isoPGF2α among wolfberry group participants suggests a physiologically relevant attenuation in oxidant burden and potential in lowering the risk of chronic diseases.

On the contrary, MDA as determined by TBARS assay, which similarly reflects lipid peroxidation, exhibited no changes in either group. Although markedly more abundant than 8-isoPGF2α, circulating MDA is more reactive and can form adducts with DNA and amino acids/proteins [38]. Moreover, it is also speculated to undergo rapid metabolic clearance [39]. Although previous shorter duration RCTs reported marked reductions in MDA following wolfberry-based interventions [12,40], its sensitivity makes it prone to fluctuations during longer intervention durations such as at present. Hence, this may yield a poorer depiction of oxidative stress status contrast to 8-isoPGF2α which exhibits greater chemical stability.

As wolfberry is one of the richest natural sources of the carotenoid zeaxanthin [41], we attempted to further elucidate its antioxidant properties, by examining the relationships between change values of the lipid peroxidation biomarkers and corresponding changes in carotenoids status. In the wolfberry group, the higher plasma zeaxanthin from weeks 8 to 16, coupled with the inverse associations with 8-isoPGF2α suggests that the zeaxanthin fraction in wolfberry may have contributed to its antioxidant properties. While the MLR also depicted inverse associations between 8-isoPGF2α with plasma β-cryptoxanthin (wolfberry group) as well as β-carotene and SCS (control group), only plasma zeaxanthin in the wolfberry group was simultaneously raised at week 16, supporting its role as a mediator for the observed change in oxidant burden. This aligns with a previous animal study which reported an attenuation of high fat diet induced oxidative stress in rats which was dosed a lutein and zeaxanthin rich marigold flower extract [42]. In addition, a 4-week clinical trial with one soft-boiled egg/d likewise reported raised plasma zeaxanthin and lutein concentrations which were negatively correlated with low-density lipoprotein oxidation [43].

Whilst able to scavenge free radicals directly, blood zeaxanthin, which is inherently lower in concentration than a majority of circulating antioxidants, may elicit its antioxidant action via alternative mechanisms of action beyond direct quenching. For instance, in an in vitro model, binding between zeaxanthin to glutathione S-transferase (GSTP1) was demonstrated to be effective in inhibiting phospholipid membrane oxidation [44]. Furthermore, zeaxanthin was also observed to destabilize kelch-like ECH-associated protein 1–nuclear factor erythroid 2-related factor 2 (Keap1-Nrf2) systems which upregulates glutathione production via the modulation of enzymes such as glutathione reductase and glutamate cysteine ligase [20,45,46]. Zeaxanthin in wolfberry was also observed to exhibit good bioavailability, as evidenced by the marked rise in plasma zeaxanthin in the wolfberry group. Present most abundantly as zeaxanthin dipalmitate in wolfberry (~90% of zeaxanthin compounds), the diester yields a structurally looser configuration over helical arrangements in its free form [47]. This enhances dissolution and thus, promotes gastrointestinal uptake. As evidenced by an earlier postprandial assessment, zeaxanthin bioavailability was demonstrated to be 23% higher following an acute administration of zeaxanthin dipalmitate over free zeaxanthin [48].

Nevertheless, besides zeaxanthin, wolfberry contains other antioxidants including LBP, polyphenols and vitamin C precursor 2-O-(β-d-glucopyranosyl) ascorbic acid [40]. The concerted action of these bioactive compounds is expected to not only further aid in the alleviation of oxidative stress, but also, support the maintenance of carotenoids status. According to the sparing hypothesis, synergism between antioxidant components may protect circulating carotenoids from oxidation, preventing stress induced depletion [49]. This is reflected in the present study which saw both marked increments in plasma carotenoids at different time points, as well as an increase in skin carotenoids deposition in the wolfberry group which reported a reduction in oxidant burden. Contrast to carotenoids in circulation, the skin is a more effective reservoir for carotenoids which makes it less sensitive to fluctuations [50]. Thus, it may yield a longer-term indication of carotenoids status for the present 16-week RCT which may explain the potential discrepancies between SCS and plasma carotenoids at different time points. Moreover in a previous cross-sectional study conducted by the same research group, it was also observed that SCS and plasma carotenoids may be independently confounded by different factors. For instance, only SCS but not plasma carotenoids were influenced by sex [51]. This was speculated to be linked to factors such as differences in body size and fat distribution [50].

Lastly, body composition was also measured as excess adiposity, particular in the truncal region is known to induce lipid peroxidation and lower antioxidant activity [52]. This is attributed a dysregulated adipokine secretion pattern and an increase in circulating free fatty acids [53,54]. Previous RCTs which investigated the impact of wolfberry fruit juice (14 days; 120 mL/d) and wolfberry fruit (45 days; 14 g/d) both deduced significant reductions in waist circumference but a lack of change in body weight [12,55]. While the present study is the first to conduct a detailed assessment of truncal adiposity using DXA, it was noted that although fat mass and lean mass were lowered and raised respectively at week 16, the changes were non-significant. This may be due to a discrepancy in adiposity contrast to the previous RCTs at baseline. Namely, the earlier studies recruited overweight and obese individuals while the present participants were within the healthy weight range. Moreover, the trial involving wolfberry fruit juice also advocated diet restrictions and exercise programs among overweight and obese subjects [55]. Nevertheless, significant time effects for total lean mass and truncal lean mass indicate considerable benefits with the adherence to the MHP. This is supported by cross-sectional evidence which found direct associations between HDP adherence and lean body mass [56]. Additionally, HDPs such as the Mediterranean diet was also reported to slow down the onset of frailty, a consequence of lean mass reduction in older adults [57,58]. Deficits in certain nutrients had been implicated to decrease lean mass [59]. For instance, in older adults, this had been linked to a lack of folate, vitamin B_12_, vitamin D, calcium as well as dietary antioxidants in general [60]. Holistically, a HDP provides diversity of nutrients to alleviate nutritional deficiencies which potentially contributes to the favorable changes in lean mass. Furthermore, according to the present changes in dietary protein (important precursors for the maintenance of lean mass) as reported previously (wolfberry: −11 (53) g/d; control: +5 (21) g/d) [61]; lower and higher intakes in the wolfberry and control groups respectively post-intervention, albeit non-significant, may explain the more prominent lean mass increase in the control group [62].

At present, limited evidence examined the impact of whole dried wolfberry consumption on oxidative stress. This is especially so amongst the middle-aged and older adults who are at a heightened vulnerability to oxidative stress. By incorporating wolfberry into a HDP, the present RCT establishes a feasible dietary strategy by taking into consideration the background dietary pattern. Moreover, this was coupled with a comprehensive assay of carotenoids status which comprised of the dietary intake as well as the concentrations in both the plasma and skin. For a carotenoid-rich food such as wolfberry, this allows for an examination of compliance as well as an assessment of carotenoid bioavailability. Finally, interpreting the correlations between changes in oxidative stress status with both the carotenoids status and body composition permits a concurrent exploration of the underlying antioxidant properties. However, caution ought to be exercised for this does not serve as a mechanistic assessment. In this aspect as well, it should be noted that the present population may also be inadequately powered to study correlations, especially among individual groups. Nevertheless, the results from the present RCT may be useful preliminary data for power calculations in the future.

Another study limitation lies in the baseline discrepancies for carotenoids status (plasma total carotenoids and lycopene) and dietary data (wholegrain intake) between groups. Although appropriate statistical techniques may be practiced for a more robust examination of the results, potential biases can still be present. Therefore, the authors recommend the use of stratified randomization for primary outcome variables to minimize heterogeneity at baseline. Additionally, while unsaturated lipids, lipoproteins and cell phospholipid membranes are key targets to oxidative stress, there are other predictor variables to assess oxidative stress which were not evaluated at present. For a more in depth evaluation of oxidative stress, the activity of enzymes which catalyze ROS reactions (e.g., superoxide dismutase and glutathione peroxidase) may be analyzed to yield a more comprehensive evaluation of antioxidant capacity. While the present study provided commercially available dried whole wolfberry, processing conditions performed prior to distribution remains unclear. As procedures such as drying had been implicated to alter the antioxidant potential of fruits and vegetables, this may be a limitation which was overlooked [63]. Nonetheless, the effects of drying technique on the antioxidant potential of wolfberry may serve as a promising direction for future research. Moreover, beyond the rich carotenoids content in wolfberry, the antioxidant properties of wolfberry, as discussed earlier, may also be synergistically enhanced by other bioactive constituents including the LBP and various polyphenols. As both constituents are metabolized by the gut microflora, the effects of wolfberry intake on the gastrointestinal microbiome and its downstream metabolic products (e.g., short chain fatty acids) can also be further investigated in future research for a more thorough elucidation of the antioxidant effects from wolfberry. Future works may also focus individuals with elevated oxidative stress such as older adults or individuals with metabolic syndrome who may benefit more substantially for the antioxidant properties of wolfberry.

## 5. Conclusions

Whole dried wolfberry intake was observed to lower of oxidant burden after a 16-week intervention and these promising changes may be driven by the rich zeaxanthin content in wolfberry. Especially among middle-aged and older adults, the incorporation of 15 g/d whole dried wolfberry to a healthy dietary pattern may serve as an effective dietary strategy to attenuate oxidative stress, a predisposing risk factor of many age-related disorders.

## Figures and Tables

**Table 1:** Changes in plasma carotenoids status at weeks 8 and 16 of randomized controlled trial ^1^.

	Study Visit	Wolfberry Group (*n* = 22) Mean (SD)	Control Group (*n* = 18) Mean (SD)	*p*	
				Time ^2^	Intervention × Time ^2^
Plasma total carotenoids (%)	Week 8	51.1 (112.1) *	−18.9 (30.7)	**0.041**	**0.008**
	Week 16	10.6 (74.6)	−35.2 (30.8)		
Plasma β-carotene (%)	Week 8	86.8 (220.7) *	−20.0 (32.2)	0.08	**0.029**
	Week 16	20.6 (130.1)	−38.3 (35.5)		
Plasma α-carotene (%)	Week 8	59.4 (145.1) *	−14.5 (30.4)	0.07	**0.034**
	Week 16	9.4 (91.6)	−28.4 (34.9)		
Plasma β-cryptoxanthin (%)	Week 8	45.7 (111.4)	−13.9 (42.7)	**<0.001**	0.05
	Week 16	7.4 (74.2)	−31.6 (36.8) *		
Plasma lutein (%)	Week 8	46.3 (78.9)	−2.5 (29.4)	**0.021**	0.45
	Week 16	14.0 (70.0)	−20.4 (34.1)		
Plasma zeaxanthin (%)	Week 8	124.2 (76.5) *	8.4 (24.7)	**<0.001**	**<0.001**
	Week 16	62.5 (91.4) *	−14.0 (29.8)		
Plasma lycopene (%)	Week 8	10.1 (101.3)	−24.7 (55.2)	0.32	0.12
	Week 16	6.5 (83.2)	−43.6 (36.9) *		

^1^ Data presented as percentage change from week 0; ^2^ Data analyzed with two-way mixed model ANOVA based on the percentage change with reference to week 0 to normalize for significant differences between plasma total carotenoids and plasma lycopene at week 0 between groups. * indicates significant difference from week 0 with post-hoc simple effects test by Bonferroni’s multiple contrasts.

**Table 2:** Associations between changes for plasma and skin carotenoids status with corresponding changes in the biomarkers of oxidative stress by intervention group.

**Wolfberry Group (*n* = 22) ^1^**	**Plasma Malondialdehyde (µmol/L) ^2^**				**Plasma 8-Iso-Prostaglandin F2α (ng/L)**			
	**Simple Linear Regression Coefficient (95% CI)**	***p***	**Multiple Linear Regression Coefficient (95% CI) ^3^**	***p***	**Simple Linear Regression Coefficient (95% CI)**	***p***	**Multiple Linear Regression Coefficient (95% CI) ^3^**	***p***
Plasma total carotenoids (µmol/L)	−0.333 (−0.885, 0.219)	0.22	−0.379 (−0.964, 0.206)	0.19	−0.204 (−0.460, 0.051)	0.11	−0.207 (−0.478, 0.063)	0.12
Plasma β-carotene (µmol/L)	−0.243 (−0.552, 0.065)	0.12	−0.289 (−0.604, 0.027)	0.07	−0.058 (−0.212, 0.096)	0.44	−0.061 (−0.222, 0.100)	0.44
Plasma α-carotene (µmol/L)	−0.342 (−0.781, 0.097)	0.12	−0.422 (−0.866, 0.022)	0.06	−0.112 (−0.328, 0.104)	0.29	−0.109 (−0.335, 0.116)	0.32
Plasma β-cryptoxanthin (µmol/L)	−0.190 (−0.760, 0.379)	0.49	−0.210 (−0.827, 0.408)	0.48	**−0.319 (−0.549, −0.089)**	**0.009**	**−0.305 (−0.555, −0.054)**	**0.020**
Plasma lutein (µmol/L)	−0.214 (−0.816, 0.388)	0.47	−0.206 (−0.897, 0.486)	0.54	−0.199 (−0.474, 0.077)	0.15	−0.285 (−0.580, 0.010)	0.06
Plasma zeaxanthin (µmol/L)	0.015 (−0.453, 0.483	0.95	0.126 (−0.382, 0.634)	0.61	−0.167 (−0.376, 0.041)	0.11	**−0.213 (−0.428, 0.002)**	**0.052**
Plasma lycopene (µmol/L)	−0.173 (−0.681, 0.334)	0.48	−0.273 (−0.912, 0.365)	0.38	−0.138 (−0.374, 0.097)	0.24	−0.087 (−0.391, 0.218)	0.56
Skin carotenoids status	0.005 (−0.003, 0.014)	0.19	0.005 (−0.004, 0.013)	0.24	0.000 (−0.004, 0.004)	0.93	0.000 (−0.004, 0.004)	0.92
**Control Group (*n* = 18) ^1^**	**Plasma Malondialdehyde (µmol/L) ^2^**				**Plasma 8-Iso-Prostaglandin F2α (ng/L)**			
	**Simple Linear Regression Coefficient (95% CI)**	***p***	**Multiple Linear Regression Coefficient (95% CI) ^3^**	***p***	**Simple Linear Regression Coefficient (95% CI)**	***p***	**Multiple Linear Regression Coefficient (95% CI) ^3^**	***p***
Plasma total carotenoids (µmol/L)	1.887 (−0.504, 4.277)	0.11	2.757 (−1.015, 6.528)	0.14	−0.526 (−1.131, 0.078)	0.08	−0.725 (−1.589, 0.139)	0.09
Plasma β-carotene (µmol/L)	1.420 (−0.698, 3.538)	0.17	1.506 (−1.471, 4.484)	0.29	**−0.610 (−1.089, −0.132)**	**0.016**	**−0.807 (−1.36, −0.259)**	**0.007**
Plasma α-carotene (µmol/L)	1.931 (−0.112, 3.974)	0.06	2.773 (−0.202, 5.747)	0.07	−0.358 (−0.914, 0.198)	0.19	−0.351 (−1.12, 0.422)	0.35
Plasma β-cryptoxanthin (µmol/L)	1.026 (−1.070, 3.123)	0.32	1.547 (−1.853, 4.946)	0.34	−0.422 (−0.931, 0.088)	0.10	−0.446 (−1.23, 0.337)	0.24
Plasma lutein (µmol/L)	**2.141 (0.096, 4.186)**	**0.041**	2.245 (−0.715, 5.204)	0.13	−0.183 (−0.776, 0.410)	0.52	−0.388 (−1.116, 0.339)	0.27
Plasma zeaxanthin (µmol/L)	1.535 (−1.012, 4.082)	0.22	1.196 (−2.037, 4.428)	0.44	−0.525 (−1.153, 0.103)	0.10	−0.652 (−1.325, 0.021)	0.06
Plasma lycopene (µmol/L)	1.489 (−0.526, 3.504)	0.14	1.945 (−0.806, 4.697)	0.151	−0.226 (−0.769, 0.316)	0.39	−0.055 (−0.757, 0.646)	0.87
Skin carotenoids status	0.006 (−0.008, 0.019)	0.38	0.005 (−0.011, 0.021)	0.52	−0.002 (−0.005, 0.002)	0.27	**−0.004 (−0.007, −0.001)**	**0.011**

^1^ Plasma carotenoids were evaluated as percentage change from week 0 to normalize for significant differences between groups at week 0; ^2^ Regression coefficient for plasma carotenoids (×10^3^); ^3^ Model adjusted for covariates age, sex and body mass index.

**Table 3:** Population anthropometric indicators and body compositions at different time points of randomized controlled trial.

	Study Visit	Wolfberry Group (*n* = 22) Mean (SD)	Control Group (*n* = 18) Mean (SD)	*p*	
				Time ^2^	Intervention × Time ^2^
Body mass index (kg/m^2^)	Week 0 ^1^	22.7 (3.7)	22.8 (2.5)	0.51	0.67
	Week 4	22.8 (3.6)	22.8 (2.3)		
	Week 8	22.8 (3.7)	22.9 (2.3)		
	Week 12	22.8 (3.7)	22.8 (2.4)		
	Week 16	22.7 (3.7)	22.9 (2.3)		
Waist circumference (cm)	Week 0	80.5 (9.6)	80.8 (7.5)	0.55	0.96
	Week 4	80.7 (10.2)	80.9 (6.6)		
	Week 8	80.4 (10.2)	80.1 (6.7)		
	Week 12	80.2 (9.9)	80.7 (7.4)		
	Week 16	80.4 (9.6)	80.2 (6.4)		
Total fat (%)	Week 0	36.5 (6.9)	35.1 (6.1)	0.08	0.44
	Week 16	36.2 (7.4)	34.4 (7.4)		
Truncal fat (%)	Week 0	37.0 (7.6)	35.7 (5.7)	0.15	0.56
	Week 16	36.7 (7.9)	35.0 (6.9)		
Appendicular fat (%)	Week 0	38.9 (8.7)	36.5 (8.9)	0.31	0.56
	Week 16	38.7 (9.7)	35.9 (10.6)		
Total fat mass (kg)	Week 0	21.3 (6.3)	20.9 (4.0)	0.12	0.62
	Week 16	21.1 (6.3)	20.5 (4.3)		
Truncal fat mass (kg)	Week 0	10.8 (3.6)	10.2 (1.9)	0.25	0.90
	Week 16	10.6 (3.5)	10.1 (2.0)		
Appendicular fat mass (kg)	Week 0	9.6 (3.0)	9.7 (2.7)	0.10	0.37
	Week 16	9.5 (3.0)	9.4 (2.9)		
Total lean mass (kg)	Week 0	34.8 (6.9)	37.0 (7.5)	**0.016**	0.26
	Week 16	35.1 (7.3)	37.8 (8.1) *		
Truncal lean mass (kg)	Week 0	17.3 (3.1)	18.0 (3.4)	**0.014**	0.25
	Week 16	17.5 (3.4)	18.5 (3.7) *		
Appendicular lean mass (kg)	Week 0	14.6 (3.6)	16.0 (3.9)	0.08	0.32
	Week 16	14.7 (3.8)	16.3 (4.2)		

^1^ Independent t-test which compared between the wolfberry and control groups indicated no significant differences in anthropometric indicators and body composition at week 0; ^2^ Data analyzed with two-way mixed model ANOVA. * indicates significant difference from week 0 with post-hoc simple effects test by Bonferroni’s multiple contrasts.

## Data Availability

The data presented in this study will be made available on request from the corresponding author. The data are not publicly available due to restrictions of informed consent and institutional guidelines.

## Appendix A

**Table A1:** Plasma carotenoids status at different time points in randomized controlled trial.

	Study Visit	Wolfberry Group (*n* = 22) Mean (SD)	Control Group (*n* = 18) Mean (SD)	*p*		
				Week 0 ^1^	Time ^2^	Intervention × Time ^2^
Plasma total carotenoids (µmol/L)	Week 0	2.02 (0.90)	2.75 (0.95)	**0.018**	**0.002**	**0.005**
	Week 8	**2.45 (0.99) ***	2.14 (0.85)			
	Week 16	1.92 (1.03)	1.68 (0.89)			
Plasma β-carotene (µmol/L)	Week 0	0.43 (0.30)	0.65 (0.40)	0.06	**0.008**	**0.049**
	Week 8	**0.54 (0.39) ***	0.50 (0.34)			
	Week 16	0.39 (0.29)	0.37 (0.27)			
Plasma α-carotene (µmol/L)	Week 0	0.26 (0.12)	0.33 (0.12)	0.05	**0.005**	**0.030**
	Week 8	**0.32 (0.16) ***	0.28 (0.13)			
	Week 16	0.24 (0.14)	0.23 (0.11)			
Plasma β-cryptoxanthin (µmol/L)	Week 0	0.45 (0.05)	0.67 (0.41)	0.05	**<0.001**	**0.014**
	Week 8	0.52 (0.36)	0.52 (0.29)			
	Week 16	0.40 (0.31)	**0.41 (0.27) ***			
Plasma lutein (µmol/L)	Week 0	0.39 (0.21)	0.37 (0.10)	0.70	**0.005**	0.10
	Week 8	0.50 (0.21)	0.35 (0.12)			
	Week 16	0.37 (0.18)	0.29 (0.14)			
Plasma zeaxanthin (µmol/L)	Week 0	0.16 (0.05)	0.15 (0.03)	0.47	**<0.001**	**<0.001**
	Week 8	**0.34 (0.10) ***	0.16 (0.03)			
	Week 16	**0.25 (0.13) *^#^**	0.12 (0.04)			
Plasma lycopene (µmol/L)	Week 0	0.33 (0.22)	0.58 (0.45)	**0.027**	**0.026**	0.12
	Week 8	0.24 (0.10)	0.33 (0.19)			
	Week 16	0.27 (0.21)	**0.26 (0.33) ***			

^1^ Data analyzed with independent t-test between groups at week 0; ^2^ Data analyzed with two-way mixed model ANOVA. Plasma carotenoids were evaluated using absolute concentrations * indicates significant difference from week 0 with post-hoc simple effects test by Bonferroni’s multiple contrasts. A comparison between week 16 absolute plasma carotenoid concentrations were additionally evaluated with analysis of covariance (ANCOVA) controlled for corresponding week 0 concentrations. ^#^ indicates statistically significant difference.

**Table A2:** Population dietary and carotenoids intake at different time points of randomized controlled trial ^1^.

Dietary Factors	Study Visit	Wolfberry Group (*n* = 21) Mean (SD)	Control Group (*n* = 17) Mean (SD)	*p*
Fruits (servings/d)	Week 0	1.7 (1.3)	2.7 (1.7)	0.06
	Week 8	3.2 (1.5)	2.5 (1.3)	0.13
	Week 16	3.4 (1.4)	2.4 (1.2)	**0.028** ^2^
Vegetables (servings/d)	Week 0	2.2 (1.8)	2.0 (1.2)	0.66
	Week 8	2.3 (0.9)	2.2 (1.4)	0.77
	Week 16	2.9 (1.4)	2.8 (1.5)	0.96
Wholegrains (servings/d)	Week 0	0.3 (0.4)	1.0 (0.8)	**0.001**
	Week 8	1.6 (1.1)	1.6 (1.0)	0.89
	Week 16	2.2 (2.3)	1.8 (1.0)	0.64
Meats and alternatives (servings/d)	Week 0	2.6 (0.8)	2.5 (1.0)	0.67
	Week 8	2.4 (0.7)	3.0 (1.0)	**0.016**
	Week 16	2.7 (1.3)	2.7 (1.2)	0.74
Total carotenoids (mg/d)	Week 0	8.63 (7.50)	11.09 (7.04)	0.31
	Week 8	9.35 (6.53)	8.94 (6.46)	0.71
	Week 16	10.51 (5.06)	12.83 (7.38)	0.34
β-carotene (mg/d)	Week 0	3.46 (3.09)	4.34 (3.12)	0.39
	Week 8	3.51 (2.65)	3.96 (3.00)	0.84
	Week 16	3.89 (2.38)	6.30 (4.75)	0.08
α-carotene (mg/d)	Week 0	0.42 (0.82)	0.59 (1.04)	0.59
	Week 8	0.47 (0.65)	0.62 (0.79)	0.69
	Week 16	0.54 (0.63)	0.80 (1.17)	0.49
β-cryptoxanthin (mg/d)	Week 0	0.11 (0.16)	0.18 (0.30)	0.43
	Week 8	0.20 (0.28)	0.20 (0.22)	0.87
	Week 16	0.23 (0.26)	0.17 (0.19)	0.45
Lutein and zeaxanthin (mg/d)	Week 0	3.10 (2.85)	3.58 (2.35)	0.58
	Week 8	3.27 (3.35)	3.43 (2.80)	0.89
	Week 16	3.49 (2.56)	4.35 (3.62)	0.45
Lycopene (mg/d)	Week 0	1.52 (2.27)	2.40 (3.46)	0.35
	Week 8	1.90 (3.30)	0.73 (0.79)	0.10
	Week 16	2.37 (2.09)	1.21 (1.49)	**0.011**

^1^ Week 0 dietary data compared with independent t-test between wolfberry and control groups. Week 8 and 16 dietary data compared with analysis of covariance (ANCOVA) controlled for corresponding week 0 intake. ^2^ *p* = 0.24 with the omission of wolfberry intake (0.5 servings/d).

**Table A3:** Associations between absolute change values of plasma carotenoids concentration and skin carotenoids status with corresponding changes in the biomarkers of oxidative stress for combined study population.

	Plasma Malondialdehyde (µmol/L) ^1^				Plasma 8-Iso-Prostaglandin F2α (ng/L)			
	Simple Linear Regression Coefficient (95% CI)	*p*	Multiple Linear Regression Coefficient (95% CI) ^2^	*p*	Simple Linear Regression Coefficient (95% CI)	*p*	Multiple Linear Regression Coefficient (95% CI) ^2^	*p*
Plasma total carotenoids (µmol/L)	0.005 (−0.027, 0.036)	0.76	0.007 (−0.030, 0.044)	0.70	**−0.015 (−0.025, −0.005)**	**0.004**	**−0.017 (−0.028, −0.007)**	**0.002**
Plasma β-carotene (µmol/L)	0.000 (−0.099, 0.100)	1.00	−0.005 (−0.117, 0.108)	0.94	**−0.044 (−0.075, −0.013)**	**0.007**	**−0.048 (−0.081, −0.015)**	**0.006**
Plasma α-carotene (µmol/L)	0.024 (−0.241, 0.288)	0.86	0.022 (−0.286, 0.330)	0.89	**−0.089 (−0.175, −0.013)**	**0.044**	**−0.100 (−0.195, −0.005)**	**0.039**
Plasma β-cryptoxanthin (µmol/L)	0.014 (−0.100, 0.129)	0.80	0.024 (−0.101, 0.150)	0.70	**−0.055 (−0.090, −0.020)**	**0.003**	**−0.055 (−0.091, −0.018)**	**0.005**
Plasma lutein (µmol/L)	0.071 (−0.113, 0.254)	0.44	0.066 (−0.139, 0.271)	0.52	**−0.066 (−0.125, −0.006)**	**0.032**	**−0.076 (−0.138, −0.013)**	**0.019**
Plasma zeaxanthin (µmol/L)	0.126 (−0.208, 0.461)	0.45	0.173 (−0.194, 0.541)	0.34	−0.104 (−0.215, 0.007)	0.07	**−0.125 (−0.239, −0.010)**	**0.034**
Plasma lycopene (µmol/L)	0.002 (−0.118, 0.122)	0.98	0.019 (−0.118, 0.156)	0.79	−0.033 (−0.073, 0.007)	0.10	−0.034 (−0.077, 0.010)	0.13
Skin carotenoids status	0.005 (−0.002, 0.012)	0.14	0.005 (−0.002, 0.012)	0.19	−0.001 (−0.004, 0.001)	0.31	−0.002 (−0.004, 0.001)	0.12

^1^ Regression coefficient for plasma carotenoids (×10^3^); ^2^ Model adjusted for covariates age, sex and body mass index.

**Table A4:** Association between change values of body compossition with corresponding changes in the biomarkers of oxidative stress by intervention group.

**Wolfberry Group** **(*n* = 22)**	**Plasma Malondialdehyde (µmol/L) ^1^**		**Plasma 8-Iso-Prostaglandin F2α (ng/L) ^1^**	
	**Simple Linear Regression Coefficient (95% CI)**	***p***	**Simple Linear Regression Coefficient (95% CI)**	***p***
Total fat (%)	−0.01 (−1.47, 2.70)	0.55	231 (−776, 1238)	0.63
Truncal fat (%)	0.57 (−2.77, 3.91)	0.72	−6 (−867, 855)	0.99
Appendicular fat (%)	−0.80 (−3.93, 2.32)	0.59	509 (−255, 1274)	0.18
Total fat mass (kg)	5.7 (−53.6, 65.0)	0.84	0.39 (−14.86, 15.65)	0.96
Truncal fat mass (kg)	6.9 (−101.4, 115.2)	0.89	4.12 (−31.88, 23.64)	0.76
Appendicular fat mass (kg)	21.4 (−108.6, 151.4)	0.73	8.73 (−24.46, 41.93)	0.59
Total lean mass (kg)	8.6 (−55.8, 73.0)	0.78	8.99 (−24.88, 6.90)	0.25
Truncal lean mass (kg)	−42.9 (−153.7, 68.0)	0.42	−4.73 (−33.69, 24.23)	0.73
Appendicular lean mass (kg)	69.3 (−36.1, 174.7)	0.18	−23.76 (−49.51, 1.98)	0.07
**Control Group** **(*n* = 18)**	**Plasma Malondialdehyde (µmol/L)**		**Plasma 8-Iso-Prostaglandin F2α (ng/L)**	
	**Simple Linear Regression Coefficient (95% CI)**	***p***	**Simple Linear Regression Coefficient (95% CI)**	***p***
Total fat (%)	1.29 (−1.04, 3.61)	0.26	396 (−732, 1523)	0.47
Truncal fat (%)	0.72 (−1.32, 2.75)	0.47	242 (−732, 1216)	0.61
Appendicular fat (%)	1.46 (−0.35, 3.27)	0.11	422 (−477, 1321)	0.34
Total fat mass (kg)	14.7 (−23.6, 53.0)	0.43	8.03 (−10.09, 26.15)	0.37
Truncal fat mass (kg)	2.47 (−62.6, 67.5)	0.94	7.12 (−23.64, 37.88)	0.64
Appendicular fat mass (kg)	47.0 (−25.7, 119.6)	0.19	17.63 (−17.51, 52.77)	0.31
Total lean mass (kg)	−16.6 (−47.6, 14.5)	0.28	−0.00 (−15.22, 15.19)	1.00
Truncal lean mass (kg)	−33.1 (−92.0, 25.7)	0.25	−2.48 (−31.38, 26.43)	0.86
Appendicular lean mass (kg)	−30.0 (−94.0, 34.1)	0.34	1.90 (−29.26, 33.07)	0.90

^1^ Regression coefficient for absolute fat and lean masses ×10^6^ for plasma malondialdehyde; ×10^3^ for plasma 8-iso-prostaglandin F2α.
